# Chest radiographic score and lactate dehydrogenase are independent risk factors linked to mortality in Middle East Respiratory Syndrome Coronavirus (MERS-CoV) patients

**DOI:** 10.1186/s43055-021-00635-6

**Published:** 2021-10-05

**Authors:** Karuna M. Das, Rajvir Singh, Khalid Al Dossari, Sandeep Subramanya, Shreesh Kumar Ojha, Taleb AlMansoori, Jamal Aldeen Alkoteesh

**Affiliations:** 1 Department of Radiology, College of Medicine and Health Sciences, UAEU, 17666 Al Ain, United Arab Emirates; 2 Department of Biostatistics, All India Institute of Medical Sciences, New Delhi, India; 3 Department of Medical Imaging, King Fahd Medical City, Riyadh, Saudi Arabia; 4 Department of Physiology, College of Medicine and Health Sciences, UAEU, Al Ain, United Arab Emirates; 5 Department of Pharmacology, CMHS, UAEU, Al Ain, United Arab Emirates; 6 Department of Radiology, Tawam Hospital, Al Ain, United Arab Emirates

**Keywords:** MERS-CoV, ARDS, LDH, Mortality, Prediction

## Abstract

**Background:**

Despite the dominance of Covid-19 in the current situation, MERS-CoV is found infrequently in the Middle East. When coupled with the chest radiographic score, serum biochemical parameters may be utilized to assess serum biochemical changes in individuals with different degrees of MERS-CoV infection and to predict death. The purpose of this study was to examine the association between increased LDH levels and severe MERS-CoV outcomes utilizing ventilation days and an elevated chest radiographic score.

**Results:**

Fifty-seven patients were included in the retrospective cohort. The mean age was 44.9 ± 13.5 years, while the range was between 12 and 73 years. With an average age of 53.3 ± 16.5 years, 18 of 57 (31.6%) patients were classified as deceased. The deceased group showed a substantially greater amount of LDH than the recovery group (280.18 ± 150.79 vs. 1241.72 ± 1327.77, *p* = 0.007). A cut-off value of > 512 LDH was established with a C-statistic of 0.96 (95% CI 0.92–1.00) and was 94% sensitive and 93% specific for mortality. Multivariate cox regression analysis revealed that log_e_ (LDH) (adjusted HR: 9.91, 95% CI: 2.44–40.3, *p* = 0.001) and chest radiographic score (adjusted HR: 1.24, 95% CI: 1.05–1.47, *p* = 0.01) were risk factors for mortality, whereas ventilation days were a protective factor (adjusted HR: 0.84, 95% CI: 0.76–0.93, *p* = 0.001).

**Conclusion:**

According to our results, blood LDH levels of > 512 had a 94% sensitivity and 93% specificity for predicting in-hospital mortality in patients infected with MERS-CoV. The chest radiographic score of 11.34 ± 5.4 was the risk factor for the mortality (adjusted Hazard ratio HR: 1.24, 95% CI: 1.05–1.47, *p* = 0.01). Thus, threshold may aid in the identification of individuals with MERS-CoV infection who die in hospital.

## Background

Global scientific interest has focused on the Coronavirus 2 Severe Respiratory Syndrome because of the current epidemic (SARS-CoV-2). An earlier zoonotic and extremely dangerous coronavirus, the Middle East Respiratory Syndrome coronavirus (MERS-CoV), is a source of worry, particularly in Saudi Arabia and surrounding nations. MERS-CoV epidemics are associated with a death rate of up to 34% [1, 2]. Until recently, 2586 MERS-CoV cases have been reported globally, with 939 fatalities [3]. Since January 2021, Saudi Arabia has recorded five MERS-CoV cases, including three fatalities [3]. Pneumonia is often present at the time of presentation, and severe infection develops, particularly in elderly patients with comorbidities [4]. Various studies have shown the diagnostic use of chest radiography, particularly radiographic scores for clinical correlation in viral lung diseases such as SARS-CoV-1 and MERS-CoV [5–7].

Primary cases have a 35% mortality rate, whereas secondary cases have a 20% mortality rate [4]. An elderly patient, male predominance, diabetes mellitus, chronic lung disease, chronic renal illness, low albumin level, and progressive lymphocytopenia were all indicators of a grim prognosis [8–10]. Lactate dehydrogenase (LDH) is found extracellularly in response to cell injury or inflammation [11]. LDH is a biomarker associated with individuals with the poorest prognosis when infected with viruses [11–13]. Elevated serum LDH levels are associated with several respiratory diseases, including CAP (Community-acquired pneumonia), Pneumocystis Jiroveci pneumonia (PJP), and Mycoplasma pneumonia [4, 13]. LDH levels were increased in individuals with active MERS-CoV infection and lung fibrosis associated with MERS [14, 15]. Although patients with severe MERS-CoV had increased serum LDH levels, no research has examined the effect on the disease's severity or death [15]. Thus, a better knowledge of the function of LDH and the relationship between the chest radiographic score and other parameters could aid in treating MERS-CoV patients. This retrospective study determines the LDH level and its correlation with chest radiographic score and their clinical significance for MERS-CoV infection severity and mortality in a Middle Eastern population.

## Methods

### Data sources and the study population

The study was authorized by the institutional review boards (IRBs) and ethics committees. Because of the retrospective nature of this research, formal informed consent was not needed. All investigations adhered to the Helsinki Declaration.

The retrospective research included 57 MERS-CoV patients with accessible chest radiographs and laboratory data who were verified by RT-PCR [16]. Between July 1, 2014, and September 13, 2019, the researcher collected 57 patients diagnosed with MERS-CoV from two separate hospitals in the Middle East. The following were the inclusion criteria: (1) serum LDH levels, and (2) acceptable chest radiographs. Those with deficient chest radiographs and lack of serum LDH levels were excluded. All medical records were surveyed, and data were abstracted before being recorded into and cross-checked in a computerized database. In this research, the investigators examined independently predicted factors, which were categorized: (1) the chest radiographic score (2) days of ventilation (3) diabetes mellitus and its Association (4) Patients' ages; (5) Gender; and (6) Laboratory values for lactate dehydrogenase (LDH), alanine transaminase (ALT), and aspartate aminotransferase (AST) that were associated with the patient's mortality. Separately, two thoracic radiologists with 12 (KD) and 22 (K.M.D) years of expertise in thoracic imaging evaluated the anonymized chest radiographs on clinical picture archive and communication system monitors. They graded the opacification of the lung parenchyma based on chest radiographs. Each lung was divided into three zones, and the extent to which each zone was involved was determined [5]. On a scale of 0 (normal) to 4 (complete involvement of one lung zone), the air space opacities caused by MERS-CoV lesions were evaluated. The results for each of the six zones in each chest radiograph sample were averaged to provide a composite chest radiographic score ranging from 0 to 24 according to the degree of lung parenchymal involvement. Disputes between the two radiologists were resolved via an agreement. Patients were divided into two groups: those who died were classed as the deceased, while those who survived were categorized as the recovery group.

### Statistical analysis

For interval variables such as age, ALT, and AST, descriptive statistics as means and standard deviations were calculated, while for categorical variables, the frequency was calculated using percentages. Student t tests were used to determine the significance of the difference between the mean levels of two groups (LDH ≤ 512 vs. LDH > 512) for interval variables, with *p*-values for equal or unequal variance according to Levene's test. The connection between the two groups and categorical variables such as gender, diabetes mellitus, and deceased, and abnormal chest x-ray was examined using chi-square or Fisher's exact tests. The correlation coefficient between the chest radiography score and the ventilation days was determined. The Kaplan–Meier survival curve was shown as a graph representing the median survival time in days. After adjusting for relevant and crucial factors, a multivariate Cox proportional hazard analysis was conducted to determine the relevance of log_e_ (LDH). Sensitivity and specificity were computed using c- statistics to determine the discriminatory ability of the LDH cut-off value. A *p*-value of 0.05 was considered statistically significant (two-tailed). SPSS 26.0 statistical software was used to perform the analysis.

## Results

**Fig. 1:**
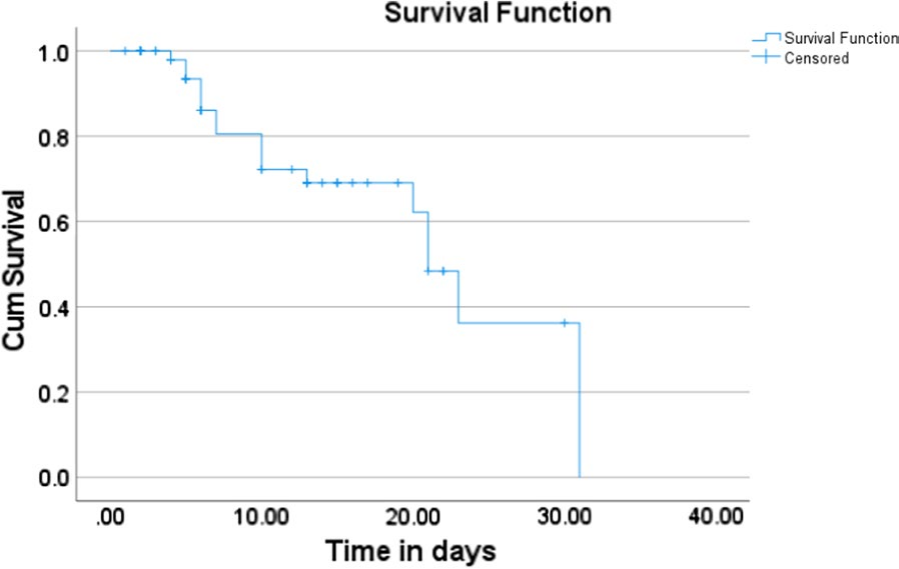
Survival curve for 57 cases who are having LDH values

The study population comprised 57 confirmed cases of MERS-CoV, ranging in age from 44.9 to 13.5 years and with a male to female ratio of 24:33. Eighteen of 57 (31.6%) patients died, with an average age of 53.3 ± 16.5 years, and 39 of 57 (68.4%) patients recovered, with an average age of 41.1 ± 9.9 years (*p* = 0.001). When compared to the recovery group, the deceased group exhibited substantially higher LDH levels (280.18 ± 150.79 vs. 1241.72 ± 1327.77, *p* = 0.007) and concurrently increased AST (56.7 ± 59.6 vs. 442.2 ± 1076.1, *p* = 0.14) and ALT (65.2 ± 561.6 vs. 124.6 ± 174.8, *p* = 0.18). Compared to the usual value, the AST and ALT were increased by 8xULN and 3xULN, respectively (ULN = upper limit of normal). Thirty-eight patients out of 57 required ICU hospitalization, with 32 needing mechanical ventilation. The median survival time was 21 days (Fig. 1). The follow–up time ranged from one day to 210 days.

**Fig. 2:**
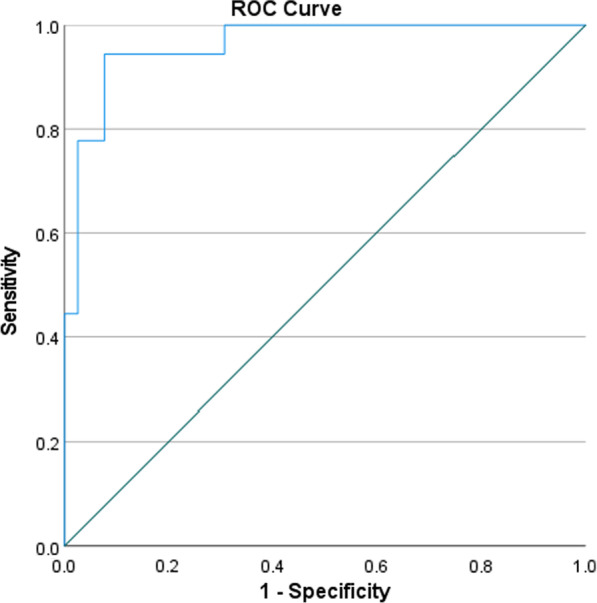
ROC curve to decide LDH level to discriminate between deceased versus recovered. It shows LDH cut-off value > 512 had 94% sensitivity and 93% specificity for the mortality showing the cut-off > 512 LDH value discriminate accurately 96% (95% CI: 92% to 100%) between deceased and recovered patients

**Fig. 3:**
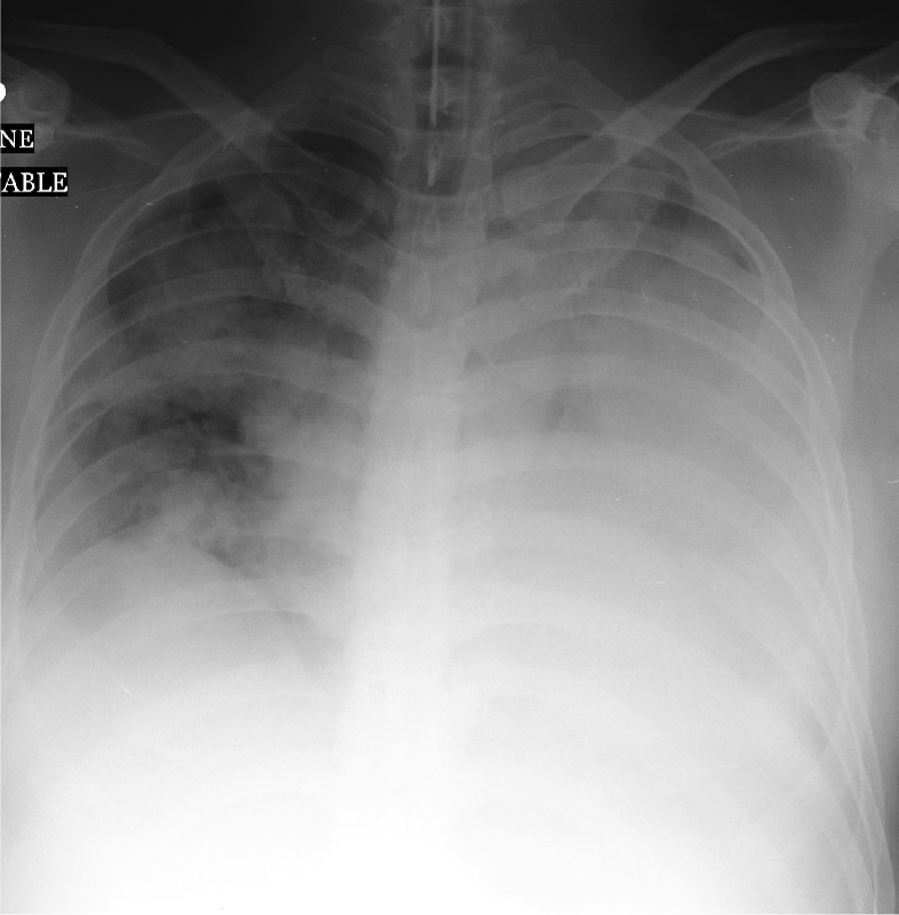
A 52-year-old man who developed Middle East respiratory syndrome coronavirus. Frontal chest radiograph obtained on day 8 shows bilateral multifocal patchy airspace disease with predominant hemiopacification of the left lung; chest radiographic score is 14 with LDH of 760. The patient died ten days after initial admission

**Fig. 4:**
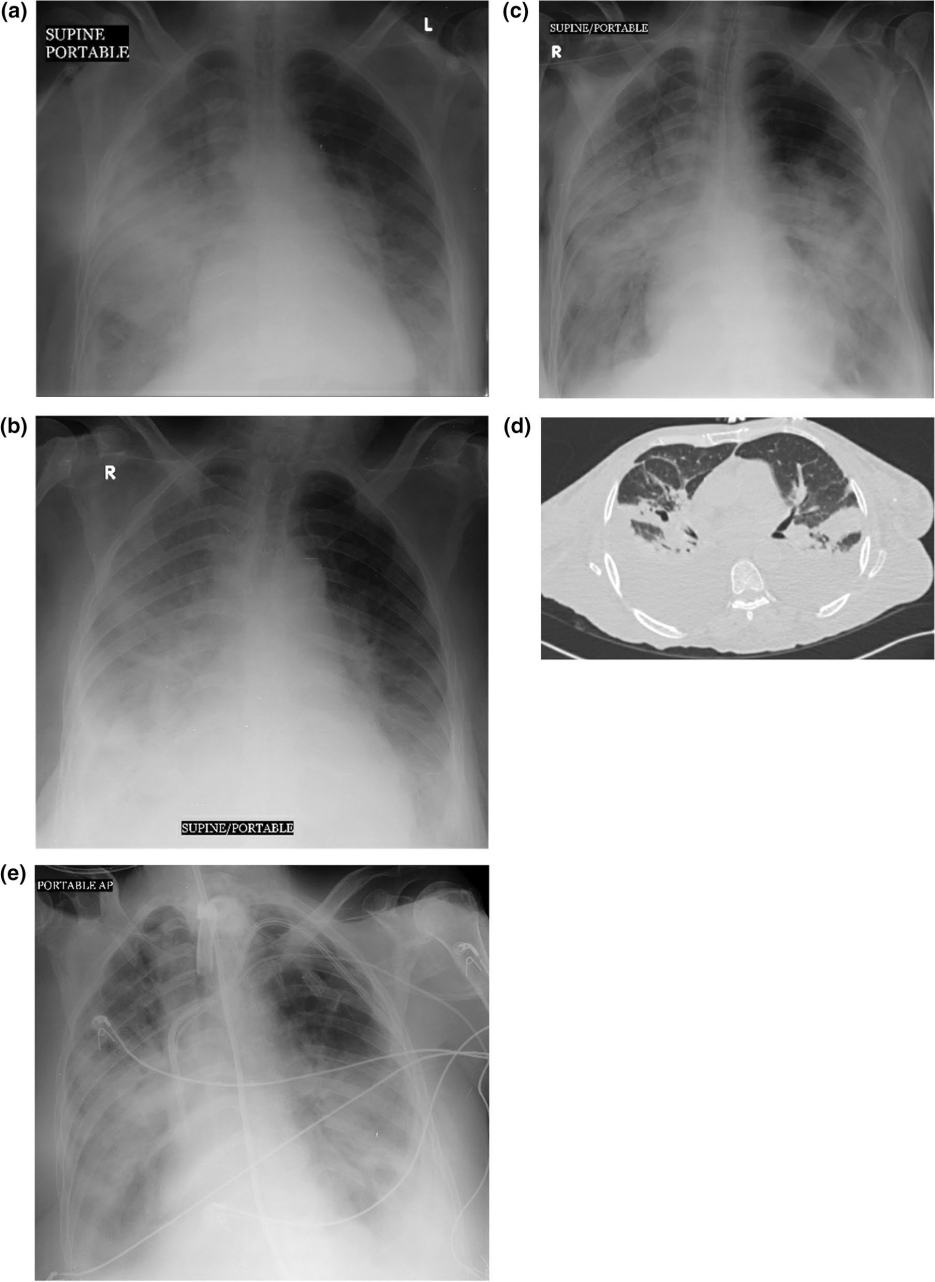
A 68-year-old man with Middle East respiratory syndrome coronavirus. Serial chest radiographs show a type 4 pattern of progression. **A** A frontal chest radiograph shows airspace disease involving the in the right upper, mid and lower zone with a chest radiographic score of 12 with bilateral pleural effusion. **B** On day 4, a follow-up frontal chest radiograph shows further deterioration of right lung air space opacity with increase of bilateral pleural effusion. **C** On day 6, further worsening of the bilateral air space opacities noted. **D** A CT scan done on the same day shows bilateral pleural effusion with bilateral air space consolidations. **E** On day 8, the patient further deteriorated and was put on ventilation. At this stage his serum LDH was 1326 and he died on day 10

The data were then examined further using the cut-off value for the LDH variable (Fig. 2). The > 512 LDH level (LDH ≤ 512 and LDH > 512 was chosen at a C-statistic of 0.96, 95% CI: 0.92–1.00) was found to be 94% sensitive and 93% specific for mortality. The cut-off value of the > 512 LDH was found to be associated with the deceased group [1 (2.7%) vs. 17 (85.0%), *p* = 0.001)]. The LDH > 512 group had a higher proportion of age in years, ventilation days, chest radiographic score, abnormal chest x-ray, and diabetes mellitus than the ≤ 512 LDH level group (Tables 1 and 2, Figs. 3, 4). Correlations between chest radiographic score and ventilation days were statistically significant (*r* = 0.44, *p* = 0.001).

**Table 1:** Comparison of clinical parameters according to LDH level in 57 MERS-Cov patients

Variable	Group statistics				
	LDH	*N*	Mean	Std. Deviation	*p-*value
AST	≤ 512	37	45.43	41.51	0.11
	> 512	20	424.55	1018.84	
ALT	≤ 512	37	54.22	49.58	0.04
	> 512	20	138.95	166.31	
Age in years	≤ 512	37	41.3	11.25	0.01
	> 512	20	51.6	14.97	
Ventilation days	≤ 512	37	2.81	6.01	0.001
	> 512	20	11.8	9.56	
Chest radiographic score	≤ 512	37	2.01	3.7	0.001
	> 512	20	11.34	5.43	
Admission days	≤ 512	37	9.22	6.85	0.05
	> 512	20	13.55	9.08

**Table 2:** Association of demographic and clinical characteristics according LDH in 57 MERS-Cov patients

Variable	LDH ≤ 512 (*n* = 37)	LDH > 512 (*n* = 20)	*p-*value
Male	17 (45.9%)	7 (35%)	0.42
Deceased	1 (2.7%)	17 (85.0%)	0.001
Chest X-ray Abnormal	21 (56.8%)	20 (100%)	0.001
Diabetes mellitus	1 (2.7%)	9 (45.0%)	0.001

Age in years, gender, ALT, AST, ventilation days, chest radiographic score, diabetes mellitus, and log_e_ of LDH (Log transformation of LDH levels were done because of skew data) were used as independent variables and deceased as the dependent variable with admission days as a time factor variable in multivariate Cox regression analysis. The analysis revealed that ventilation days were a protective factor (adjusted HR: 0.84, 95% CI: 0.76–0.93, *p* = 0.001), while the chest radiographic score (adjusted Hazard ratio HR: 1.24, 95% CI: 1.05–1.47, *p* = 0.01) and log_e_ (LDH) (adjusted HR: 9.91, 95% CI: 2.44–40.3, *p* = 0.001) were risk factors for mortality (Fig. 2). There was no significant relationship between ALT, AST, age, or gender, and mortality in this investigation (Table 3).

**Table 3:** Multivariate cox regression analysis to see association of mortality in 57 MERS-Cov patients

Variable	Adjusted HR ratios	95% CI	*p-*value
Age in years	0.99	0.95–1.03	0.6
Ventilation days	0.84	0.76–0.93	0.001
Chest radiographic score	1.24	1.05–1.47	0.01
Diabetes mellitus	1.01	0.24–4.24	0.99
LN (LDH)	9.91	2.44–40.30	0.001
Gender: Male	0.58	0.13–2.66	0.49

## Discussion

MERS-Cov spread across the Middle East, causing human illness via solitary cases, community clusters, and nosocomial outbreaks. This retrospective research showed a link between an elevated serum LDH level and death in MERS-CoV patients. According to ROC analysis, serum LDH levels of > 512 throughout the illness were 94% sensitive and 93% specific for death and were linked with the deceased group [1 (2.7%) vs. 17 (85.0%), *p* = 0.001)]. The Cox proportional hazard model showed that increased serum log_e_ (LDH) levels during admission days were an independent predictor of MERS-CoV severity and mortality (adjusted HR: 9.91, 95% CI: 2.44–40.3, *p* = 0.001). We observed that individuals with higher chest radiographic scores (adjusted HR: 1.24, 95% CI: 1.05–1.47, *p* = 0.01) were more likely to have a fatal outcome, as shown by the Cox proportional hazards model.

Although deceased individuals had a significantly higher mean age (54.37 ± 16.68, *p* = 0.001), age was not a risk factor after adjusting for other multivariate cox regression analysis factors (adjusted HR: 0.99, 95% CI: 0.95–1.03; *p* = 0.60). Our present observation was contrary to previous experience that older age is associated with increased risk of mortality in MERS-CoV patients [17]. One reason our deceased cohort's mean age was lower (54.37 ± 16.68, *p* = 0.001) than in previous series with higher range of mortality [17]. Ahmed et al. found that patients aged ≥ 60 years had a significantly higher death rate (45.2 percent vs 20.0 percent; *p* = 0.001) when compared to patients aged < 60 years [18]. Diabetes mellitus (adjusted HR: 1.01, 95% CI: 0.24–4.24, *p* = 0.99) and chest radiographic score (adjusted HR: 1.24, 95% CI: 1.05–1.47, *p* = 0.01) were risk factors that could be interpreted as increasing the likelihood that the problem will deteriorate further and exacerbate the burden of the current condition. Similarly, after adjusting for other relevant factors, it was found that LN (Log Natural) LDH was a very significant risk factor for mortality (adjusted HR: 9.91, 95% CI: 2.44–40.30, *p* = 0.001). Increased ventilation days are rated as protective in multivariate cox regression analysis (adjusted HR: 0.84, 95% CI: 0.76–0.93, *p* = 0.001), which can be interpreted to mean that an increase in ventilation days may confer resilience to patients undergoing critical care, decreasing the likelihood of a fatal outcome.

According to Assiri et al., 49% of their MERS-CoV patients exhibited increased LDH levels, compared to 50%–71% of global SARS-CoV-1 cases; however, no particular level was noted in MERS-CoV patients who died [15]. Ghamadi et al. found that 62.7 percent of their MERS-CoV patient groups had increased LDH > 300 U/L, with 56.3 percent surviving and 43% dying [19]. They have not referred to a cut-off point between survivors and those who have died. Serum LDH levels > 512 were 94% sensitive and 93% specific for disease-related death in our population. A recent pandemic of SARS-CoV-2, a SARS-CoV-2 virus that resembles MERS-CoV, has also been associated with substantially increased LDH levels [20]. Chang et al. discovered that a serum LDH cut-off value of 359.50 U/L accurately predicted SARS-CoV-2 mortality with 93.8% specificity and 88.2% accuracy [20]. Higher LDH levels were an independent risk factor for SARS-CoV-2 severity (adjusted HR: 2.73, 95% CI: 1.25–5.97, *p* = 0.012) and mortality (adjusted HR: 40.50, 95% CI: 3.65–449.28, *p* = 0.003) according to logistic regression analysis and the Cox proportional hazards model [20]. We do not know why MERS-CoV and SARS-CoV-2 have different ROC values, but we believe MERS-CoV has a greater fatality rate and more severe respiratory symptoms than SARS-CoV-2 [21, 22]. SARS-CoV-2, SARS-CoV-1, and MERS-CoV are linked with a mortality rate of 6.76%, 9.6%, and 35.5%, respectively [21, 22].

In our current series, the chest radiography score for the deceased group was considerably higher (13 ± 2.6 5 vs.8 ± 5.6, *p* = 0.001) than for the recovered group. Significant inverse relationships between radiography scores and SaO2 have been reported [7, 23]. All patients with diffuse consolidation required extra ventilation days [7]. We discovered a strong correlation between the chest radiographic score and the number of ventilation days in our current sample (*r* = 0.44, *p* = 0.001).

Although SARS-CoV-2, SARS-CoV-1, and MERS-CoV all frequently infect the liver, various complicating variables may skew the interpretation of existing data about the association between infection and hepatocyte damage [24]. The serum aminotransferases AST and ALT are two of the most critical enzymes for diagnosing liver illness or injury. The AST level is less specific for the liver than for the ALT level. Duan et al. reported that about 38% of SARS-CoV-1 patients exhibited elevated ALT activity, with higher levels linked to more severe disease [25]. The average increase in ALT and AST activity was 3 xULN and 1.5 xULN, respectively, in MERS patients [10]. Increased ALT and AST levels were seen in 3xULN and 8xULN of the present cohort of deceased MERS-CoV patients, respectively. We do not know what is driving our population's disproportionate rise in AST. Increased AST levels, but not ALT levels, were significantly higher in people with SARS-CoV-2 who had gastrointestinal symptoms than in those who did not (29.35 vs. 24.4, *p* = 0.02) [24]. We did not associate the hematological markers with the clinical presentation of the patients in this group.

Notably, the chest radiographic characteristics of 99 patients were previously reported in four separate studies [5, 26–28] focusing solely on the radiological aspects of MERS-CoV pneumonia in chest radiographs, from which 57 cases with LDH levels were selected for the present study. Therefore, the present study examines the relationship between laboratory parameters and chest radiographic score in MERS-CoV pneumonia patients to determine if LDH is associated with increased mortality risk.

## Limitation

One of the most significant drawbacks of our study was the small sample size. As a result, differentiating between genuine and random effects is difficult. Excluding individuals who do not have LDH findings may add to the bias toward more severe cases. Since the research was retrospective, patients lacked appropriate laboratory measurements and follow-ups. We found death in 18 of 57 patients, and statistical bias may have influenced our results owing to the limited sample size. The hazard ratio's 95 percent confidence interval is higher than the study's inadequate sample size. The research lacks external validation owing to the scarcity of data.

## Conclusion

Like SARS-CoV-1 and SARS-CoV-2, MERS-CoV has pandemic potential and continues to show itself in a people as a sporadic illness. According to our findings, serum LDH levels of > 512 had a sensitivity of 94% and a specificity of 93% for predicting in-hospital mortality in patients infected with MERS-CoV. Diabetes mellitus and a high chest radiographic score were risk factors, suggesting that their presence raises the likelihood that the illness would worsen and aggravate the burden of the pre-existing condition. These objective indicators may identify patients with a good prognosis. These markers may aid in the identification of patients who need additional rigorous therapy. To generalize the findings, a large cohort study with a sufficient sample size may be performed.

## Data Availability

Yes.

## Abbreviations

MERS: Cov-Middle East respiratory syndrome coronavirus
LDH: Lactate dehydrogenase
CAP: Community-acquired pneumonia
ALT: Alanine transaminase
AST: Aspartate aminotransferase
ULN: Upper limit of normal; HR-Hazard Ratio
LN: Log Natural
SARS-CoV-2: Severe acute respiratory syndrome coronavirus 2
SARS-Cov-1: Severe acute respiratory syndrome coronavirus-1
RT-PCR: Reverse transcription Polymerase chain reaction
ARDS: Adult respiratory distress syndrome
